# Human and Livestock Surveillance Revealed the Circulation of Rift Valley Fever Virus in Agnam, Northern Senegal, 2021

**DOI:** 10.3390/tropicalmed8020087

**Published:** 2023-01-27

**Authors:** Moufid Mhamadi, Aminata Badji, Mamadou Aliou Barry, El Hadji Ndiaye, Alioune Gaye, Mignane Ndiaye, Moundhir Mhamadi, Cheikh Talibouya Touré, Oumar Ndiaye, Babacar Faye, Boly Diop, Mamadou Ndiaye, Mathioro Fall, Andy Mahine Diouf, Samba Niang Sagne, Cheikh Loucoubar, Hugues Fausther-Bovendo, Ara, Amadou Alpha Sall, Gary Kobinger, Ousmane Faye, Mawlouth Diallo, Oumar Faye

**Affiliations:** 1 Virology Department, Institut Pasteur de Dakar, Dakar 12900, Senegal; 2 Parasitology Department, Université Cheikh Anta Diop de Dakar, Dakar 10700, Senegal; 3 Medical Zoology Department, Institut Pasteur de Dakar, Dakar 12900, Senegal; 4 Epidemiology, Clinical Research and Data Science Department, Institut Pasteur de Dakar, Dakar 12900, Senegal; 5 Institut Pasteur de Dakar, DIATROPIX, Dakar 12900, Senegal; 6 Ministry of Health and Social Action, Dakar 12900, Senegal; 7 Ministry of Livestock and Animal Production, Dakar 12900, Senegal; 8 Global Urgent and Advanced Research and Development, Batiscan, QC G0X 1A0, Canada; 9 University of Texas Medical Branch, Galveston, TX 77555-0132, USA

**Keywords:** Rift Valley Fever Virus, survey, humans, livestock, northeastern Senegal

## Abstract

The mosquito-borne disease caused by the Rift Valley Fever Virus (RVFV) is a viral hemorrhagic fever that affects humans and animals. In 1987, RVFV emerged in Mauritania, which caused the first RVFV outbreak in West Africa. This outbreak was shortly followed by reported cases in humans and livestock in Senegal. Animal trade practices with neighboring Mauritania suggest northern regions of Senegal are at high risk for RVF. In this study, we aim to conduct a molecular and serological survey of RVFV in humans and livestock in Agnam (northeastern Senegal) by RT-PCR (reverse transcription real-time polymerase chain reaction) and ELISA (Enzyme-Linked Immunosorbent Assay), respectively. Of the two hundred fifty-five human sera, one (0.39%) tested RVFV IgM positive, while fifty-three (20.78%) tested positive for RVFV IgG. For animal monitoring, out of 30 sheep recorded and sampled over the study period, 20 (66.67%) showed seroconversion to RVFV IgG antibodies, notably during the rainy season. The presence of antibodies increased significantly with age in both groups (*p* < 0.05), as the force of RVF infection (FOI), increased by 16.05% per year for humans and by 80.4% per month for livestock sheep. This study supports the usefulness of setting up a One Health survey for RVF management.

## 1. Introduction

Rift Valley Fever Virus (RVFV) is an arthropod-borne virus belonging to the *Phlebovirus* order in the *Phenuiviridae* family which infects humans and animals, including animal livestock. The virus is transmitted mainly by infected mosquito bites during a blood meal, yet humans can also acquire RVFV infection through contact with an infected animal or handling of contaminated animal products, as well as in the healthcare setting during the care of infected patients [1].

RVF was first documented in 1930 in the Rift Valley region of Kenya in East Africa [2]; the disease has since been reported throughout Africa and the Arabic peninsula, and the diffusion is likely attributed to livestock trade. In 1987, RVFV emerged in Mauritania and caused the first RVFV outbreak in West Africa [3]. Due to the high risk of RVF introduction by livestock trade with neighboring Mauritania, Senegal’s northern regions are the most exposed. In Senegal, serological and molecular evidence of RVFV circulation has been reported in humans, livestock, and mosquitoes [4,5,6,7,8,9,10,11]. *Aedes vexans*, *Culex quinquefasciatus,* and *Culex poicilipes* can transmit the disease [12].

In humans, after infection and a few days of incubation, clinical signs and symptoms such as fever, headache, backache, vertigo, anorexia, and photophobia appear. In a few cases, additional severe clinical manifestations can appear, including hepatitis, jaundice, further neurological disease, and hemorrhagic disease [13]. RVFV infects a wide range of animals with signs including fever, bloody diarrhea, and abortion, as well as behaviors indicative of listlessness, loss of appetite, disinclination to move, and abdominal pain [13]. A differential influence of age on mortality was reported, with juveniles more susceptible to severe outcomes than adults [13]. RVFV remains detectable by reverse transcription real-time polymerase chain reaction (RT-PCR) 10 days after symptom onset. Antibody levels start to increase at day four (4) after symptom onset for immunoglobulin M (IgM) and on day seven (7) after symptom onset for immunoglobulin G (IgG). These antibodies remain detectable by serological assay for at least forty-two (42) days for IgM and several years for IgG [14]. 

The RVFV genome is a ribonucleic (RNA) genome that is composed of three segments: small (S), medium (M), and large (L). The S segment encodes the nucleocapsid (N) protein and non-structural proteins (NS), which are translated from overlapping open reading frames (ORF). The M segment encodes for the glycoproteins (Gn and Gc) and a non-structural protein (NSm). Lastly, the L segment encodes the RNA-dependent RNA polymerase (RDRP) [15].

Several commercialized vaccines are available to protect against RVFV infection, including the live attenuated MP-12 and the inactivated vaccine TSI-GSD-200 for human immunization [16], as well as the ancestral live attenuated vaccine Smithburn strain and the newly developed live attenuated vaccine Clone 13 for livestock animal vaccination [17]. Even so, thus far, no specific pharmaceutical treatment for RVF exists, which prompts an urgency to assess the circulation of the disease. Here we used a One Health site to assess the circulation of RVFV in Agnam, an area near Mauritania at risk of infection in animals and humans related to the importation of infected livestock.

## 2. Materials and Methods

### 2.1. Study Sites

**Figure 1:**
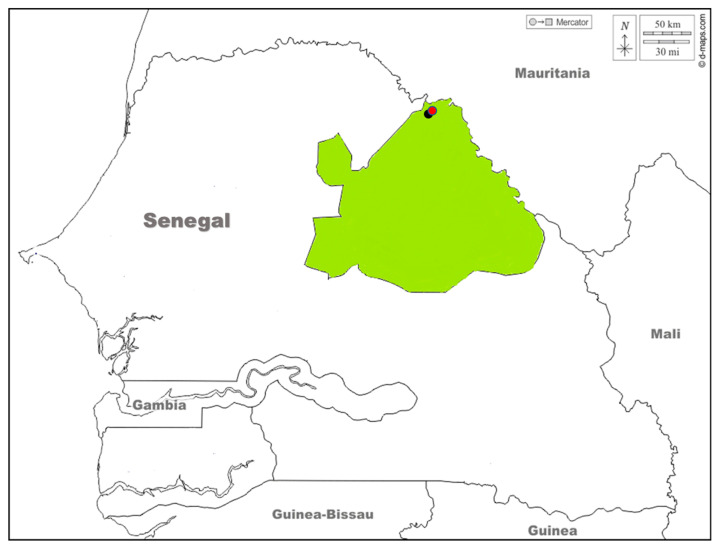
Study sites (Idite for animal survey represented by the black dot and Agnam Civol for human survey represented by the red dot) located in Matam (colored in green) in Northern Senegal. Download from (https://d-maps.com/carte.php?num_car=25271&lang=fr, accessed on 7 January 2023) and edit.

This survey was established in the Matam region (15°06′18″ N and 13°38′30″ W), more particularly in Agnam, in the villages located around Agnam Civol (16°00′18″ N, 13°41′35″ W) and Idite (15°55’09.5” N 13°43’05.1” W) (Figure 1). These Sudano-Sahelian areas are located in the northeast of the country on the border of Mauritania with an estimated population of 654,951 inhabitants. Within Agnam, Agnam Civol constitutes the urban environment, which includes markets and hospitals. In contrast, Idite contains pastoral activities such as grazing and livestock breeding.

In Agnam, the rainy season starts in June and ends in October, with a low amount of 369 mm of rainfall per year.

### 2.2. Blood Sampling in Humans and Animals

During this study, to assess the potential circulation of RVFV in Agnam, human and animal samples were collected.

Every febrile patient who signed their consent was blood sampled in the Agnam Civol healthcare sentinel site between June 2021 and December 2021 as already described [18]. Blood samples were transiently conserved at +4 °C then transferred to the arbovirus and viral hemorrhagic fever virus unit at the Institut Pasteur de Dakar as part of the ongoing Syndromic Sentinel Surveillance network in Senegal (4S network) [19]. In the laboratory, arboviral and viral hemorrhagic fever infections were tested by RT-PCR (reverse transcription real-time polymerase chain reaction) and ELISA (Enzyme-Linked Immunosorbent Assay) for the research of the antibodies immunoglobulin M (IgM) and immunoglobulin G (IgG).

For the animal survey, 30 sheep located in Idite (15°55’09.5” N 13°43’05.1” W) were blood sampled by jugular routes and tested for RVFV infection by RT-PCR and anti-RVFV IgG by ELISA in February 2021 to include individuals without any contact with the virus. Only the sheep that tested negative for RVFV infection were included in this subsequent study. These sheep were blood sampled every 2 weeks up to day 56 and then monthly from February to December 2021 to monitor RVFV infection. The sheep were also monitored for typical signs of RVFV infection, symptoms such as abortion and an increased mortality rate in young animals.

### 2.3. Serological Assay for RVFV

The presence of anti-RVFV immunoglobulin M (IgM) was tested in human sera with an in-house immunocapture Enzyme-Linked Immunosorbent Assay (ELISA) [18]. Goat anti-human IgM (Sera care, Milford, MA, USA) was diluted at 1/1000 in carbonate–bicarbonate buffer (0.015 M sodium carbonate, 0.035 M sodium bicarbonate, pH 9.6) as coating buffer. Coated plates (Immulon II 96-well microtiter plates; Dynatech Industries, Inc., Rockdale, Australia) were incubated overnight at 4 °C. After the incubation step, the plates were washed thrice using phosphate-buffered saline (PBS, Gibco, Ph 7.4) 1X at 0.05% Tween 20. Sera and homologous positive and negative antibody controls were added to the wells at a dilution of 1:100. The diluent used was phosphate-buffered saline with 0.05% Tween 20 and 1% non-fat dry milk. After one-hour incubation at 37 °C and 3 washing steps, viral and normal antigens of infected suckling mouse liver obtained from the WHOCC collection were added at dilutions of 1:100. After one-hour incubation at 37 °C and 3 washing steps, polyclonal Mouse Ascitic Fluids specific to RVF Ag were prepared at 1:1000 in diluent solution. Plates were incubated for a further 1 h at 37 °C and washed 3 times. Peroxidase-conjugated anti-mouse IgG horseradish was subsequently added at a dilution of 1/30,000 in the diluent buffer.

After one-hour incubation at 37 °C and 3 washing steps, 3, 3′, 5, 5′-tetramethylbenzidine (TMB SIGMA Aldrich, USA) was used as a substrate and the reaction was stopped 5 min later with 1 N sulfuric acid. Reactions were measured using a MultiSkan microplate reader (Thermofisher Scientific, Waltham, MA, USA) at an absorbance of 450 nm with 620 nm as a passive reference.

The difference (ΔDO) was measured between the optical density yielded by the sample viral antigen OD and the sample negative antigen OD; any ΔDO > 0.2 was considered positive and any lower value was considered negative.

For anti-RVFV IgG detection in human and animal sera, we used an indirect in-house Enzyme-Linked Immunosorbent Assay (ELISA) test [18]. Plate wells were sensitized by adding 100 μL per well of 50 ng of RVF glycoprotein (Abcam) diluted in phosphate-buffered saline (phosphate-buffered saline (PBS, Gibco, Ph 7.4)) 1X and incubated overnight at 4 °C. Plate wells were then washed 3 times with a washing buffer composed of PBS 1X and 0.005% Tween 20. After this washing step, the antigen residues were blocked by adding 200 µL per well of a blocking buffer composed of PBS 1x, 0.05% Tween 20, and 5% non-fat skimmed milk. After 1 h of incubation at 37 °C, the plate wells were washed thrice with the washing buffer, and 1/100 diluted sera and antibody controls (negative and positive) were added to the plate wells. Then, the plates were incubated at 37 °C for 1 h. After a washing step, 100 μL of a species-specific antibody conjugated with horseradish peroxidase (rabbit anti-sheep IgG (Biorad) or a goat anti-human IgG (KPL)) was added to each well, then the plates were incubated for 1 h at 37 °C. The Tetramethylbenzidine (TMB) substrate was added after three final washing steps, and then the reaction was stopped with 1 N sulfuric acid. The plate well was read using the absorbance 450/620 filters using a Multiskan microplate reader, and the generated data were processed in Microsoft Excel. RVFV glycoprotein ELISA cut-off was determined with a finite mixture model.

### 2.4. Reverse Transcription Real-Time Polymerase Chain Reaction for RVFV

Ribonucleic acid (RNA) was extracted from human and sheep sera sampling using the QIAamp RNA Viral Kit (Qiagen GmbH, Heiden, Germany). Briefly, RNA was extracted from 140 μL of sera using AVL lysing buffer and eluted in 60 μL using AVE buffer according to the manufacturer’s instructions. Then, extracted RNA was immediately stored at −80 °C until further use. The presence of the RVFV virus non-structural (NSs) gene of the small segment was tested by RT-PCR using the AgPath-ID One-step RT-PCR kit (Thermofisher) mixed with primers (forward primer TGCCACGAGTYAGAGCCA and reverse primer GTGGGTCCGAGAGTYTGC) and probe (probe TCCTTCTCCCAGTCAGCCCCAC), as previously described [20]. The reaction mixture consisted of 5 μL RNA, 12.5 μL of buffer, 4 μL of RNase-free water, 1 μL of each primer at 10 μm, 0.5 μL of the probe at 10 μm, and 1 μL of enzymes for a total volume of 25 μL. The RVFV NSs gene detection was performed on QuantStudio 5 (Applied Biosystems, Foster City, CA, USA) thermocycler platform using the following cycling conditions: reverse transcription step at 50.0 °C for 10 min, denaturation at 95.0 °C for 15 min, 40 cycles’ hybridization for 15 s at 95.0 °C, and elongation for 1 min at 60 °C.

### 2.5. Statistical Analysis

Data were processed in Microsoft Excel. Data analysis was carried out in R software version 4.1.1 (2009–2022 RStudio, PBC). The risk of RVFV exposure was evaluated by logistic regression with statistical significance set up as *p* < 0.05. The rate at which susceptible individuals become infected, which is called the force of infection (FOI), was evaluated as previously described [21]. The FOI is an important public health parameter for measuring the weight or the burden of disease and the effect of outbreak management programs. The analysis of the human survey (one serosurvey) replicated the methodology of the Colombo survey as previously described [21]. Alternatively, the animal survey (multiple serosurveys) replicated the methodology of the Medellin survey as previously described [21].

We use the finite mixture model to determine the cut-off of RVFV glycoprotein ELISA assay. Finite mixture models are classes of statistical models for addressing the heterogeneity of data and characterizing the unobserved class of each observation. It is well-studied in the field of statistics and has a wide range of applications in the fields of biostatistics, bioinformatics, medical care, and computer science. Particularly, the finite mixture model can be used to model bimodal data and derive a cut-off value that separates the two peaks. In this case, the main task of data analysis is clustering, i.e., using numerical vectors (IgG concentration, Optic Density (OD) ELISA) to identify positive and negative samples. We can model such a bimodal distribution by a finite mixture model that uses continuous distributions from the exponential family [22]. In the absence of clear expectations on the shape of these distributions, we can consider the normal, the gamma, and the Weibull distributions. In this paper we use the normal distribution. The normal distribution is defined on all real numbers and is characterized by 2 parameters: a location parameter μ and a scale parameter σ. The first parameter accounts for the location of most of the data and corresponds to the mean of the normal distribution. The second parameter accounts for the spread of the data around the location parameter and corresponds to the standard deviation. We refer to μ1 and σ1 for the location and scale parameters of the peak of the lower optic density (OD ELISA) and μ2 and σ2 for the location and scale parameters of the peak of the higher optic density (OD ELISA). The density of the bimodal distribution of optic density ELISA thus reads as follows.

f(x|λ,μ1,σ1,μ2,σ2)=λ×D1(x|μ1,σ1)+(1−λ)×D2(x|μ2,σ2)

The parameters of the finite mixture model are estimated by the expectation maximization algorithm [23] as coded by the function. These parameters include two parameters for each of the two probability distributions and a mixture parameter.

We use the fitted finite mixture model to identify a cut-off value that discriminates the two modes of the dataset. For that, we compute the probability for a datum to belong to distribution D1 as

$$p1=\frac{\lambda \times D1\left(x|\mu 1,\sigma 1\right)}{\lambda \times D1(x|\mu 1,\sigma 1)+\left(1-\lambda \right)\times D2(x|\mu 2,\sigma 2)}$$

and the probability for a datum to belong to distribution D1 as

$$p2=\frac{\left(1-\lambda \right)\times D2(x|\mu 2,\sigma 2)}{\lambda \times D1(x|\mu 1,\sigma 1)+\left(1-\lambda \right)\times D2(x|\mu 2,\sigma 2)}$$

We equate this probability to the type I error we aim at to find the cut-off value.

## 3. Results

### 3.1. Human Survey

During the survey, 255 human sera were collected from febrile patients between June 2021 and December 2021. The median temperature was 38.5 °C with a sex ratio of 0.84 (46.27% for men and 53.73% for women). The patient’s median age was 19 years, with a minimum age of 2 months and a maximum age of 98 years. Analysis of the signs and symptoms shows that headache (91.78%) was the most documented sign/symptom followed by myalgia (65.72%), arthralgia (30.87%), asthenia (7.36%), and rashes (5.66%). The most sampled age group was 0–20 years, and the most febrile patients sampled were in October (81/255 or 31.76%).

One febrile patient tested positive for a recent RVFV infection by testing positive for the anti-RVFV IgM test. We noticed that 53 were positive for anti-RVFV IgG, and none of the collected human sera were positive by RVFV NSs RT-PCR test. The anti-RVFV IgM-positive patient was a 60-year-old woman from Bagonde village located in Idite, and she had contact with livestock animals. She had a consultation 2 days after the symptoms’ onset. Her clinical signs and symptoms were headache and arthralgia with a temperature of 38.6 °C, and her malaria histidine rich protein (HRP) rapid diagnostic test (RDT) was negative. The anti-RFVV IgG detection test performed 20 days later was positive, and the patient regained good health without any complications. We also noticed that 30 years old was the median age of the positive tested (IgM and IgG) patients, with comparable rates between women and men (sex ratio = 0.89). The overall seroprevalence (IgM + IgG) was 21.17% in this study. 

**Figure 2:**
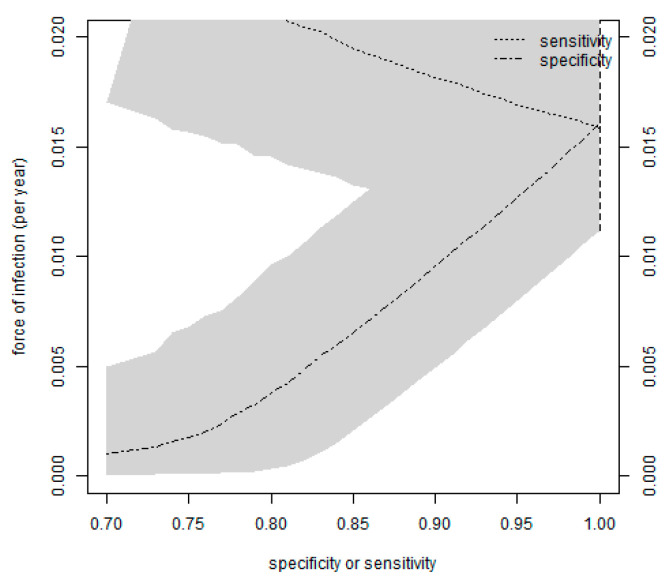
The force of infection among Agnam’s tested human population is calculated for each value of sensitivity or specificity, which is considered to be fixed. Figure 2 shows that the 95% credible intervals are represented in the grey zones. When specificity reaches 100% sensitivity is less than 100%, with the converse also being true. On the right side of the plot of Figure 2, as accuracy approaches 100%, the credible interval approaches the statistical significance of 5% from the standard binomial (vertical dashed line).

We estimated the force of infection to be 16.05% per year with a credible interval of 11.22 to 22.11%, with sensitivity and specificity both varying (Figure 2).

The seropositivity rates between men and women did not show a statistical difference. Still, the RVFV exposure or infection risk becomes significatively higher with increasing age (*p*-value = 0.027), and anti-RVFV immunoglobulins (IgM + IgG) rates were significatively higher in Bele village (Table 1), which is a pastoral area like Idite.

### 3.2. Animal Survey

**Figure 3:**
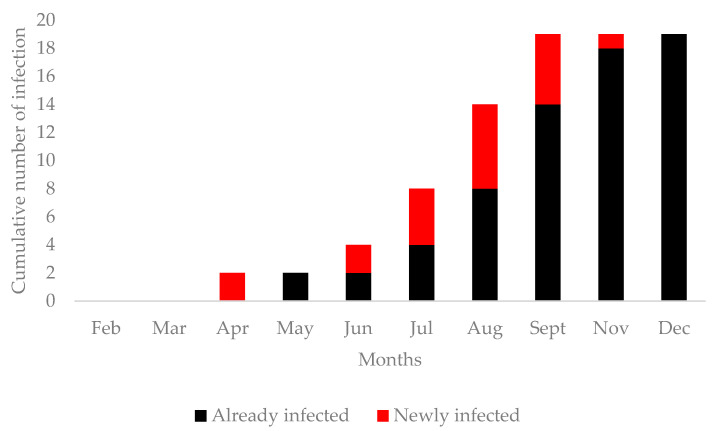
Temporal occurrence of anti-RVFV IgG among Sheep.

From February to December 2021, 314 sera were sampled from a study group of 30 sheep located in Idite. This population principally consisted of females (94.11%), and the ages ranged from a minimum of four (4) months to a maximum of eighteen (18) months, with a median age of eight (8) months. The analysis shows that 20 of the 30 (66.67%) animals seroconverted to RVFV during the eleven (11) monthly surveys (Figure 3). 

Furthermore, the typical clinical signs of RVFV infection, such as abortion storm among pregnant females, were not observed in the sentinel sheep. Additionally, mortality rates did not abnormally increase among lambs or young sheep during the livestock sheep survey. Reverse transcription real-time polymerase chain reaction (RT-PCR) for RVFV NSs detection shows that no sheep was positive for RVFV infection. We noticed that RVFV seroconversion occurred mostly in the rainy season (*p* = 0.0152), particularly in August (*p* = 0.002786) and September (*p* = 0.000191). The presence of anti-RVFV IgG was significatively higher in adult sheep (*p* = 0.0000000387) (Table 2). During the survey, two deaths were recorded, but neither was positive for RVFV by RT-PCR and Enzyme-Linked Immunosorbent Assay (ELISA) tests (Table 2).

**Figure 4:**
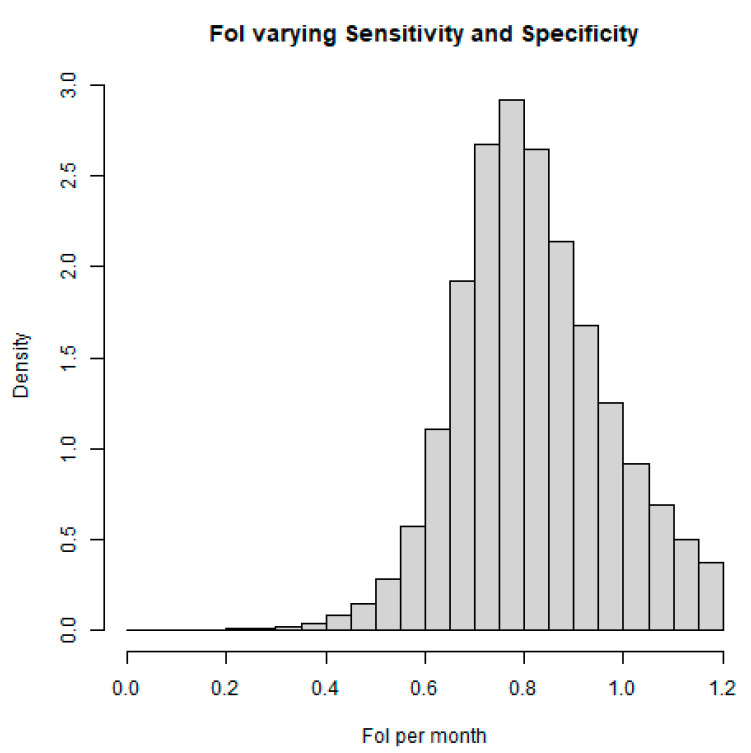
Force of infection (FOI) among livestock sheep. The density is determined by the proportion of individuals (sheep) contained in each age group.

Analysis shows that the force of infection (FOI) increased by 80.4% per month (CI: 53.6 to 100%) (Figure 4).

## 4. Discussion

Rift Valley Fever Virus (RVFV) remains a major threat to human and livestock health. To survey RVFV emergence, a molecular and serological pilot survey study of RVF was undertaken in Agnam, a northern Senegal area with a high risk of RVFV emergence. Among the two hundred fifty-five human patients tested, one (1) was positive for anti-RVFV immunoglobulin M (IgM) (0.39%), and the anti-RVFV IgG test performed 20 days after the anti-RVFV IgM test became positive. To our knowledge, this patient represents the first ever reported case of acute human RVFV infection in the Agnam area and shows the usefulness of monitoring emerging disease surveillance through a human survey. The anti-RVFV IgM patient reported having contact with livestock animals such as cattle, goats, sheep, horses, and donkeys, which classed her at high risk of RVF infection or exposure. Ultimately, this woman recovered without complications. Importantly, our results show that 53 (20.78%) patients presented anti-RVFV IgG, which means they were already exposed to RVFV. This finding shows that RVFV actively circulates among the human population in this area. Human overall seroprevalence (IgM + IgG) is 21.17%, which is a comparable rate to the 21.30% found in Rosso (northern Senegal) [24], the 22.30% found in Yonofere (central Senegal) [25], and the 21% found in Moudjeria (Mauritania) [26]. However, the 20.78% rate is lower than the seroprevalence reported in Kenya [27], at a rate of 36.40%. This difference could be explained by the fact that during the Kenyan study, they sampled a high-risk population, unlike our study where only febrile patients were tested. Still, our seroprevalence is higher than those found in Nigeria (5.3% [28]) and in Senegal (6.5% in Linguere (central Senegal), Mbour (western Senegal), and Kedougou (southern Senegal) [29], 0.89% in Kedougou (southern) [30], and 5.2% in Diawara (southern Senegal) [31]). This difference could be due to the different epidemic factors or profiles between our study areas. Historically, northern Senegal including Agnam is known to be at high risk for RVFV emergence because of its proximity to endemic areas. Furthermore, Agnam is located in the Matam region, one of the main paths of human transport of livestock into Senegal, Mauritania, and Mali [32]. Interestingly, previous studies show that transboundary livestock trade between countries has been related to the Comoros island RVFV outbreak [33] and the reemergence of RVFV in Somalia [34]. Indeed, epizootics (amplification of the virus in an animal population such as livestock animals) acting as a vector have usually preceded human RVFV epidemics [22].

The animal survey showed that seroconversion to RVFV occurred in 20 of the 30 livestock sheep (66.67%), and anti-RVFV IgG persisted until the end of the study (December 2021). Furthermore, high mortality rates among the sheep, including abortion storms, were not observed during the livestock animal survey. These results could indicate that RVFV circulates sub-clinically in our livestock sheep. The high seroprevalence (66.67%) found in this study is comparable to the 75% reported in Senegal [29], 55% from Mauritania [35], and 45% from Uganda [34]. Our prevalence is higher than that reported in Senegal (14.3%) [8], Mauritania (3.8%) [36], the Central African Republic (12.9%) [37], and Uganda (4%) [38], which could be explained by the long exposure time (11 months) of the sentinel sheep in natural settings. The increasing seroprevalence in sheep (Figure 3) has preceded the only anti-RVFV IgM human case found in this study (Figure 2), which, in conjunction with her contact with livestock, suggests RVFV replication in sheep could have also led to the first human acute case of RVFV.

Our results show that seropositivity was significatively higher in the older population in both humans and sheep, which supports previously documented studies [16,22,38]. A study conducted in the lower Senegal river basin demonstrated that children born after the 1987 RVF outbreak have a small anti-RVFV IgG antibody rate (5%) compared to the elder population (25.3%) [22]. Our age-associated results are supported by the increased FOI with time (years or months in each group). The first recorded human acute case of RVF in Agnam is a 60-year-old woman in our study whose disease was detected in October 2021, which is in line with age and rainfall being major factors in RVF epidemiology. Likewise, nearly all seroconversion occurred in adult animals and during the rainy season (28 of 30 animals).

## 5. Conclusions

This study provided evidence of RVF circulation in humans and livestock. These results highlighted the importance of RVFV surveys in both humans and animals for averting potential RVF outbreaks. Our findings show that in Agnam, the highest RVFV seroconversion rates occur in the rainy season, and the elder population is the most at risk of RVFV exposure. Accordingly, an immunization campaign for RVFV vaccine implementation should be established in the dry season. Lastly, to improve RVFV epidemiological studies in Agnam, a survey of mosquitoes/vectors and other hosts needs to be conducted alongside human and animal surveys for the elucidation of the mechanisms underlying RVFV emergence in the northern Senegal area and beyond.

## Figures and Tables

**Table 1:** Risk factors of RVF infection among the human study group. N IgG (%) = number of people with anti-RVFV IgG antibodies (percentage of people with anti-RVFV IgG).

	N IgG (%)	OR (CI, 95%)	*p*-Value
**Sex**			
Male	25 (47.17%)	1.04 (0.56, 1.91)	0.883
Female	28 (52.83%)		
**Age**			
	NA	1.02 (1.00, 1.04)	0.027
**Season**			
Rainy	36 (67.92%)	1.36 (0.72, 2.63)	0.348
Dry	17 (32.08%)		
**Months**			
June	4 (7.54%)	1.27 (0.28, 5.22)	0.736
July	2(3.77%)	1.91 (0.22, 13.74)	0.515
August	8 (15.09%)	0.65 (0.22, 2.00)	0.445
September	7 (13.20%)	0.69 (0.21, 2.20)	0.534
October	17 (32.07%)	0.76 (0.29, 2.08)	0.548
November	9 (16.98)	0.60 (0.20, 1.80)	0.356
December	6 (11.32%)	0.55 (0.16, 1.81)	0.335
**Location**			
Agnam Civol	22 (41.50%)	1.75 (0.63, 5.69)	0.308
Agnam Godo, Agnam Sinthou Cire, Badiya, Idite, Ngouloum, Thilogne, Yero Yabe	1 (1.88%)	8.50 (0.04 6.32)	0.888
Agnam Ouro Ciré	13 (24.52%)	2.94 (0.98, 1.00)	0.063
Bagonde	5 (9.43%)	2.61 (0.63, 1.09)	0.176
Bele	4 (7.54%)	6.80 (1.26, 3.89)	0.024
Toulel Thiale	2 (3.77%)	1.51 (0.19, 8.40)	0.652

**Table 2:** Risk factors for RVF in livestock sheep. N IgG (%) = sheep with anti-RVFV IgG antibodies (percentage of sheep with anti-RVFV IgG).

	N IgG (%)	OR (CI, 95%)	*p*-Value
**Age**			
Juvenile	2 (10%)	1.21 (1.14, 1.31)	0.0000000387
Adult	18 (90%)		
**Season**			
Dry	3 (15%)	1.84 (1.12, 3.03)	0.0152
Rainy	17 (85%)		
**Months**			
February	0	1.38 (7.96, 1.33)	0.988
March	0	1.38 (2.19, 6.91)	0.988
April	2 (10%)	2.13 (3.86, 1.62)	0.401
May	0	1.00 (1.14, 8.75)	1
June	2 (10%)	2.13 (3.86, 1.62)	0.401
July	4 (20%)	4.14 (9.12, 2.94)	0.0915
August	6 (30%)	1.12 (2.75, 7.62)	0.002786
September	5 (25%)	2.02 (5.03, 1.38)	0.000191
November	1 (5%)	2.02 (5.03, 1.38)	0.000191
December	0	2.02 (5.03, 1.38)	0.000191
**Sex**			
Male	0 (0%)	6.39 (NA, 1.30)	0.985
Female	20 (100%)		

## Data Availability

Not applicable.
